# Patient Attitudes to Tonsillectomy

**DOI:** 10.1155/2012/735684

**Published:** 2012-12-24

**Authors:** Kishan Ubayasiri, Ravi Kothari, Lisha McClelland, Mriganka De

**Affiliations:** Department of Otolaryngology, Royal Derby Hospital, Uttoxeter New Road, Derby DE22 3NE, UK

## Abstract

*Introduction*. Recent changes to primary care trusts' Procedures of Limited Clinical Value (PLCV) policy mean that otolaryngologists must now follow policy rather than exercising clinical judgment when listing patients for tonsillectomy. *Objectives*. To gauge perception within the general public of when tonsillectomy is acceptable and to compare this to the current policy. *Method*. All patients or their parents attending the adult and paediatric outpatient ENT departments were asked to anonymously complete questionnaires. *Results*. One hundred and twenty-five completed questionnaires were collected. Thirty-one percent of respondents thought tonsillectomy should be offered solely on patient request, 19% after one to three bouts, and 35% after four to six bouts of tonsillitis. Only 9% thought the current guidelines were reasonable. Patients who had suffered recurrent tonsillitis or had undergone previous tonsillectomy generally thought tonsillectomy advisable after more bouts of tonsillitis than those who had not. Fourteen patients fulfilled the SIGN guidelines for tonsillectomy for recurrent tonsillitis. Of these, 13 (93%) felt that suffering 4–6 bouts of tonsillitis was reasonable before tonsillectomy. *Conclusion*. All patients we surveyed who meet the current PLCV and SIGN guidelines regarding the appropriateness of tonsillectomy for recurrent tonsillitis perceive that they are excessive, believing that 4–6 bouts of recurrent tonsillitis are adequate to justify tonsillectomy.

## 1. Introduction

Healthcare budget cuts and greater scrutiny of health economics have led to a restriction in the provision of surgical services. Many Primary Care Trusts have presented their ENT departments with a policy for “procedures of limited clinical value” (PLCVs) [1]. For our ENT department this equates to strict criteria for listing patients for tonsillectomy [2]. This is somewhat juxtaposed to the government's desire for patient choice with clinicians “putting patients at the heart of the NHS” [3]. Since many ENT operations relate to quality of life, evidence is required that these operations are still necessary and in demand, despite being regarded as PLCVs. 

The aim of this research is to gauge perception in the general public of when tonsillectomy is acceptable and to compare this to the current policy. 

## 2. Method

All patients or their parents, in the case of young paediatric patients, attending the adult and paediatric outpatient ENT departments were asked to anonymously complete questionnaires (see the appendix). The questionnaire had previously been piloted on a small sample of medical and nonmedical staff. The questionnaire results were then consolidated and analyzed.

## 3. Results

Out of 150 questionnaires, 125 (83%) completed forms were returned: 49 adult and 76 paediatric. Forty-four percent (*n* = 55) of respondents were male and 66% (*n* = 70) were female. The mean age of patients, for which questionnaires were completed, was 24 years (range 14 weeks to 83 years). 

**Figure 1:**
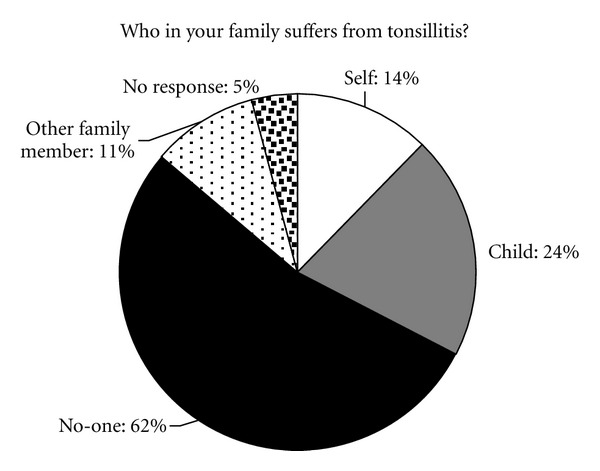


When asked whether anyone in the family was affected by tonsillitis, 14% (*n* = 17) replied “themselves”, 24% (*n* = 28) replied “their child”, and 63% (*n* = 74) replied “no-one”. 11% (*n* = 14) replied that another family member suffered from tonsillitis (Figure 1). 15% (*n* = 19) of respondents had previously undergone tonsillectomy.

**Figure 2:**
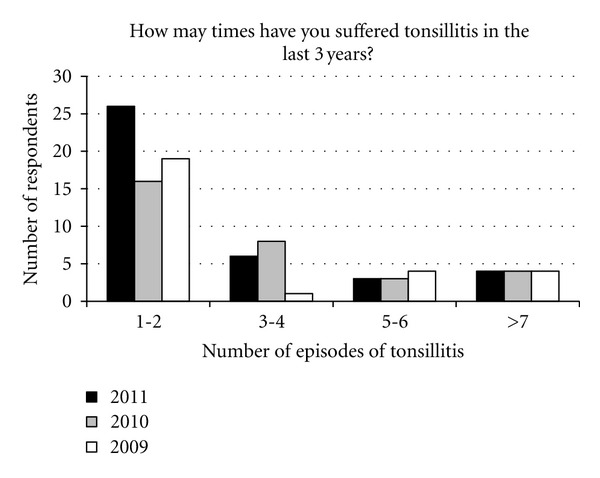


In the previous 12 months, 67% (*n* = 84) had suffered no tonsillitis, 21% (*n* = 26) 1-2 bouts, 5% (*n* = 6) 3-4 bouts, 2% (*n* = 3) 5-6 bouts, and 3% (*n* = 4) more than 7 bouts (Figure 2).

Thirty-four percent (*n* = 42) of patients had been prescribed oral antibiotics within the last 3 years for tonsillitis: 20% (*n* = 25) on 1-2 occasions, 7% (*n* = 9) on 3-4 occasions, 2% (*n* = 3) on 5-6 occasions, and 4% (*n* = 5) on more than 7 occasions.

Three percent (*n* = 8) had attended hospital for acute tonsillitis within the previous 3 years. Of these, 5 individuals attended on 1-2 occasions and 3 attended on 3-4 occasions. Of these 8 patients, 5 had required admission for analgaesia and intravenous antibiotics.

Five percent (*n* = 6) of respondents had suffered one previous quinsy and 2% (*n* = 2) had suffered 2 or more quinsies.

**Figure 3:**
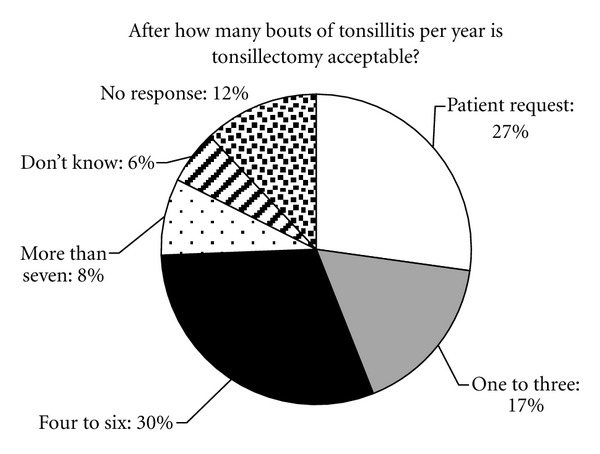


Twenty-seven percent (*n* = 34) of respondents thought that tonsillectomy should be offered on patient request, 17% (*n* = 21) after 1 to 3 bouts, and 30% (*n* = 38) after 4 to 6 bouts of tonsillitis. Only 8% (*n* = 10) thought that the current guideline of 7 or more episodes in the previous year was reasonable (Figure 3).

Patients who had suffered recurrent tonsillitis or had undergone previous tonsillectomy generally thought that tonsillectomy was advisable after more bouts of tonsillitis (4–6 bouts) than those who had not. 

In particular, 14 patients (11%) fulfilled the SIGN guidelines for tonsillectomy after recurrent tonsillitis. 4 of these patients had suffered over 7 bouts on tonsillitis in the previous year, 4 had suffered 5 episodes of tonsillitis per year for the last 2 consecutive years, and 6 had suffered 3 episodes of tonsillitis per year for the last 3 consecutive years. 

**Figure 4:**
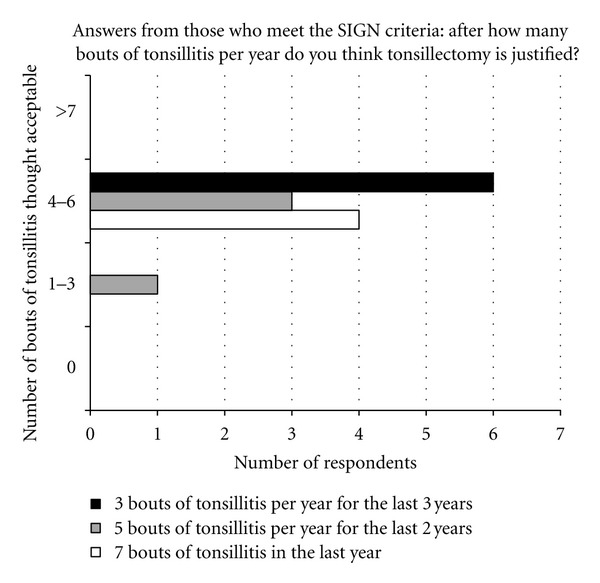


Of the 14 patients fulfilling the SIGN criteria for tonsillectomy, 93% (*n* = 13) felt that suffering 4–6 bouts of tonsillitis in a year was a reasonable indication for tonsillectomy, whilst the remaining respondent thought 1 to 3 bouts was acceptable (Figure 4).

## 4. Discussion

Primary Care Trusts receive funding to commission health services for their resident population. They have a responsibility to seek the greatest possible health advantage for local populations using allocated resources. Commissioning involves specifying, securing, and monitoring services that are of high quality, evidence-based, cost effective, meet the needs of individuals and provide “value for money in the use of public resources”.

Historically, otolaryngologists have used clinical judgment combined with local and national guidelines, GP opinion and patients wishes when considering tonsillectomy for recurrent tonsillitis. However, otolaryngologists must now follow the PLCV policy rather than exercising clinical judgment. 

Most patients with sore throat in the community do not seek primary care help [4]. A UK study of 516 women aged 20–24 years found that only one in 18 episodes of sore throat led to a GP consultation [5]. Once referred to ENT, patients undergoing a preferred treatment option for recurrent tonsillitis experence improved outcomes. Patient treatment preference tends to be influenced by medical advice and recent experience rather than age or socioeconomic status [6].

The current Scottish Intercollegiate Guidelines Network (SIGN) state the following recommended indications for consideration of tonsillectomy for recurrent acute sore throat in both children and adults [7].

- Sore throats are due to acute tonsillitis.
- The episodes of sore throat are disabling and prevent normal functioning.
- 7 or more well documented, clinically significant, adequately treated sore throats in the preceding year or …
- 5 or more such episodes in each of the preceding 2 years or …
- 3 or more such episodes in each of the preceding 3 years.

These widely accepted criteria form the basis of policies such as the PLCV document, but are based on levels 3 and 4 evidence [7, 8]. They take no account of whether the condition is improving or worsening and make little distinction between adults and children, in whom the disease may behave differently. They also take no account of the educational and psychosocial impact on children missing many weeks from school or adults who cannot work due to either their child or their own illness [9].

The limited information regarding adult sore throat and the effect of tonsillectomy, although not scientifically robust, suggests that surgery is beneficial [10]. Again, despite limited evidence in the paediatric age group, many non-controlled studies suggest tonsillectomy benefits children prone to recurrent tonsillitis, in terms of reduction of the number of sore throats and an improvement in their general health and well-being [11–13]. Furthermore, rates of patient and parental satisfaction with the outcome of tonsillectomy are in excess of 90% [14–16].

## 5. Conclusion

Enforcement of the PLCV policy restricts the use of clinical judgment, GP opinion and patients' wishes when considering patients for tonsillectomy. The PLCV policy is highly prescriptive, rigorously applying the SIGN recommendations preventing their use as guidelines, and not accommodating extenuating circumstances.

All patients we surveyed who met the PLCV and SIGN guidelines for tonsillectomy for recurrent tonsillitis perceived them excessive, with 13 of these 14 individuals believing that 4–6 bouts of recurrent tonsillitis in the previous year are adequate to justify tonsillectomy.

## Supplementary Material

The following questionnaire is designed to assess patient attitudes to tonsillectomy. Please tick whether you are completing the form for your child or yourself and then enter the age and gender of the patient concerned. Please mark your views by placing a tick in the appropriate box.

## Appendix

### Attitudes towards Tonsillectomy

The following questionnaire is designed to assess patient attitudes to tonsillectomy. Please tick whether you are completing the form for your child or yourself and then enter the age and gender of the patient concerned. Please mark your views by placing a tick in the appropriate box. Thank you.

Are you filling this form in for:

□ Yourself
□ Your child?

Age: …

Sex (M/F): …

(1) Who in your family suffers from tonsillitis?
  □ You
  □ Your child
  □ Other family member (please state) …
  □ No-one
(2) How many times have you/your child suffered from tonsillitis in 2011?
  □ None
  □ 1-2
  □ 3-4
  □ 5-6
  □ 7 or more
(3) How many times have you/your child suffered from tonsillitis in 2010?
  □ None
  □ 1-2
  □ 3-4
  □ 5-6
  □ 7 or more
(4) How many times have you/your child suffered from tonsillitis in 2009?
  □ None
  □ 1-2
  □ 3-4
  □ 5-6
  □ 7 or more
(5) How many times in the last *3 years* have you/your child come to hospital because of tonsillitis? How many of these visits have required admission?
  Visits to hospital:
    □ None
    □ 1-2
    □ 3-4
    □ 5-6
    □ 7 or more
  Hospital admissions:
    □ None
    □ 1-2
    □ 3-4
    □ 5-6
    □ 7 or more
(6) How many times in the last *3 years* have you/your child required antibiotics for tonsillitis?
  □ None
  □ 1-2
  □ 3-4
  □ 5-6
  □ 7 or more
(7) If you/your child were admitted to hospital, what treatment did you receive? Please tick as many as appropriate.
  □ Pain relief
  □ Intravenous antibiotics
(8) Have you or your child ever had a quinsy?
  □ Never
  □ Once
  □ Twice or more
  □ Don't know
(9) Have you or your child had a tonsillectomy?
  □ Yes
  □ No
(10) After how many bouts of tonsillitis per year do you think it is acceptable to have your tonsils removed (tonsillectomy)?
  □ 7 or more
  □ 4–6
  □ 1–3
  □ None

Thank you for completing this questionnaire.

The Trust adopts guidelines as to when tonsillectomy is appropriate (SIGN guidelines). The purpose of this study is to gauge patient attitudes to these and to assess the impact of tonsillitis on patients' everyday lives.
