# Supplementary material for: Patient Attitudes to Tonsillectomy
Source: Int J Family Med. 2012 Dec 24;2012:735684. doi: 10.1155/2012/735684 (PMC3540685; doi:10.1155/2012/735684)
Supplement: Supplementary file 1 — The following questionnaire is designed to assess patient attitudes to tonsillectomy. Please tick whether you are completing the form for your child or yourself and then enter the age and gender of the patient concerned. Please mark your views by placing a tick in the appropriate box. [file 735684.f1.doc]

**ATTITUDES TOWARDS TONSILLECTOMY**

The following questionnaire is designed to assess patient attitudes to tonsillectomy. Please tick whether you are completing the form for your child or yourself and then enter the age and gender of the patient concerned. Please mark your views by placing a tick in the appropriate box. Thank you.

**Are you filling this form in for: ☐ Yourself ☐ Your child?**

**Age:……………. Sex (M/F):…………..**

| 1. Who in your family suffers from tonsillitis? | | ☐ You | | | | | | | | | | | | ☐ Your child | | | | | | | | | |
| --- | --- | --- | --- | --- | --- | --- | --- | --- | --- | --- | --- | --- | --- | --- | --- | --- | --- | --- | --- | --- | --- | --- | --- |
|  |  | ☐ Other family member (please state)………………. | | | | | | | | | | | | ☐ No-one | | | | | | | | | |
| 1. How many times have you/your child suffered from tonsillitis in 2011? | ☐None | | | | ☐ 1-2 | | | | | | ☐ 3-4 | | | | | | ☐ 5-6 | | | | ☐7 or more | | |
| 1. How many times have you/your child suffered from tonsillitis in 2010? | ☐None | | | | ☐ 1-2 | | | | | | ☐ 3-4 | | | | | | ☐ 5-6 | | | | ☐ 7 or more | | |
| 1. How many times have you/your child suffered from tonsillitis in 2009? | ☐None | | | | ☐ 1-2 | | | | | | ☐ 3-4 | | | | | | ☐ 5-6 | | | | ☐ 7 or more | | |
| 1. How many times in the last **3 years** have you/your child come to hospital because of tonsillitis? How many of these visits have required admission? | Visits to hospital: | | | | | | | ☐None | | | | ☐1-2 | | | ☐3-4 | | | | ☐5-6 | | | | ☐7 or more |
|  | Hospital admissions: | | | | | | | ☐None | | | | ☐1-2 | | | ☐3-4 | | | | ☐5-6 | | | | ☐7 or more |
| 1. How many times in the last **3 years** have you/your child required antibiotics for tonsillitis. | ☐None | | | | | | ☐1-2 | | | ☐3-4 | | | | | | ☐5-6 | | | | | | ☐7 or more | |
| 1. If you/your child were admitted to hospital, what treatment did you receive? Please tick as many as appropriate. | | | | | | | | | | | | | | ☐Pain relief | | | | | ☐ Intravenous antibiotics | | | | |
| 1. Have you or your child ever had a quinsy? | | | ☐ Never | | | | | | ☐ Once | | | | ☐ Twice or more | | | | | | | ☐ Don’t know | | | |
| 1. Have you or your child had a tonsillectomy? | | | | ☐Yes | | | | | | | | | | | | | | ☐ No | | | | | |
| 1. After how many bouts of tonsillitis per year do you think it is acceptable to have your tonsils removed (tonsillectomy) | | | | | | ☐ 7 or more | | | | | | | | | | | | ☐ 4-6 | | | | | |
|  |  |  |  |  |  | ☐ 1-3 | | | | | | | | | | | | ☐ None | | | | | |

**Thank you for completing this questionnaire.**

The Trust adopts guidelines as to when tonsillectomy is appropriate (SIGN guidelines). The purpose of this study is to gauge patient attitudes to these and to assess the impact of tonsillitis on patients’ everyday lives.
